# Horn-Shaped Perforator Flaps for Plantar

**DOI:** 10.3390/jcm15093197

**Published:** 2026-04-22

**Authors:** Zhuoran Wang, Xinyi Li, Xiaojing Li, Fei Zhu, Yun Bai, Hui Cheng

**Affiliations:** 1 Department of Clinical Medicine, Anhui Medical University, Hefei 230032, China; 2545011869@stu.ahmu.edu.cn (Z.W.); 2345011727@stu.ahmu.edu.cn (H.C.); 2 Department of Plastic Surgery, The First Affiliated Hospital of Anhui Medical University, Hefei 230022, China; yfy130377@fy.ahmu.edu.cn (X.L.); yfy130381@fy.ahmu.edu.cn (F.Z.); yfy1302218@fy.ahmu.edu.cn (Y.B.)

**Keywords:** foot, skin, skin neoplasms, surgical flaps, perforator flaps

## Abstract

**Objective:** To investigate the method and clinical outcomes of employing plantar propeller perforator flaps for the repair of defects in the plantar region. **Methods:** This was a retrospective case series of 40 patients (20 males, 20 females; age range 20–75 years) who underwent plantar defect reconstruction using the horn-shaped perforator flap technique between January 2020 and October 2025. Defect etiologies included malignant melanoma (*n* = 24), melanocytic nevus (*n* = 3), and refractory wounds (*n* = 13). Defect sizes ranged from 2 cm × 1.5 cm to 5 cm × 5 cm. The primary outcome was flap survival; secondary outcomes included functional recovery (American Orthopaedic Foot and Ankle Society AOFAS score), sensory recovery (Semmes–Weinstein monofilaments), and time to full weight-bearing. **Results:** Complete flap survival was achieved in 38/40 patients (95%). Two patients (5%) experienced minor distal wound dehiscence and necrosis, successfully managed with full-thickness skin grafting and dressing changes without compromising final outcomes. Mean follow-up was 14.2 ± 6.8 months (range 3–24 months). Mean AOFAS score was 91.3 ± 5.6, with 80% achieving excellent functional recovery. Protective sensation was present in 87.1% of the tested patients. Mean time to full weight-bearing was 6.4 ± 1.8 weeks. No local tumor recurrence occurred in melanoma patients during follow-up. **Conclusions:** The horn-shaped perforator flap provides a reliable source of homologous glabrous skin for reconstructing small-to-medium-sized plantar defects while avoiding skin grafting at the donor site. Its combined rotation–advancement mechanism, flexible triangular leading-edge strategies, and preservation of multiple perforators contribute to favorable functional and aesthetic outcomes. Prospective comparative studies with standardized plantar-specific outcome measures are warranted.

## 1. Introduction

The sole, a vital weight-bearing area of the human body, is distinguished by its tough skin texture, strong wear resistance, and high-pressure tolerance [1]. In recent years, the incidence of plantar lesions, especially malignant melanoma, has been rising rapidly at an annual rate of 3–5% [2]. Consequently, the reconstruction of plantar defects has emerged as a central concern and a formidable challenge in plastic and reconstructive surgery. Given the unique characteristics and functions of plantar skin, achieving satisfactory repair of its defects is often an arduous task. Traditional axial pattern flaps are associated with complex surgical procedures, potential damage to named arteries during flap harvest, and the introduction of new donor-site morbidity [3,4,5]. For plastic surgeons, a long-standing goal has been to leverage the limited skin mobility of the sole for repair using homologous adjacent tissue, aiming for both functional restoration and an aesthetically pleasing outcome [6]. Definition of the “horn-shaped” concept and statement of innovation: Throughout this manuscript, we use the term “horn-shaped perforator flap” to describe our technique. This replaces earlier descriptors (“arc-line,” “angular,” “propeller”) to provide consistent nomenclature. The name derives from the curvilinear geometry with a widened leading edge that resembles a horn. Unlike conventional perforator propeller flaps that rely on pure rotational movement around a single skeletonized perforator, our horn-shaped flap incorporates three distinctive features: Geometric novelty: A curvilinear design with a widened leading edge (the “horn” configuration) that exceeds the defect diameter, with a total flap length roughly 3–4 times the defect length. Biomechanical novelty: A combined mechanism of rotation (45–90°) plus advancement (1–3 cm), rather than pure rotation around a single pivot point. We acknowledge that our flap functions predominantly as an advancement–rotation flap rather than a true propeller flap (which typically requires 90–180° rotation around a skeletonized perforator). Vascular handling novelty: Preservation of 2–3 perforators within a broad subcutaneous pedicle, rather than isolating and skeletonizing a single dominant perforator. This reduces the risk of pedicle torsion and venous congestion. Since January 2020, we have developed and implemented homologous adjacent plantar propeller (“arc-line”) pedicled perforator fasciocutaneous flaps for the repair of small-to-medium-sized plantar defects. This technique is indicated in clinical cases such as limb salvage after trauma, pressure ulcer reconstruction, tumor resection defects, and chronic wound coverage, especially when conventional flaps may result in excessive tissue bulk or inadequate contour adaptation. Compared with classical flaps, horn-shaped perforator flaps preserve more of the perforator vessels, reduce donor site morbidity, and allow for more flexible design to match complex wound geometries. In contrast, traditional flaps often require larger tissue harvest and may lead to higher rates of partial necrosis or contour deformity. The main purpose of using the horn-shaped perforator flap is to provide a reliable source of homologous glabrous skin for reconstructing small-to-medium-sized plantar defects, preserving weight-bearing function, and minimizing donor-site morbidity. Our method focuses on the effective integration of perforator-based propeller flaps and local advancement flaps and has demonstrated promising clinical results.

## 2. Materials and Methods

### 2.1. Clinical Data

This study was carried out in the Department of Plastic Surgery at the First Affiliated Hospital of Anhui Medical University, spanning from January 2020 to October 2025. This is a retrospective observational study. The study group comprised 40 patients, evenly split into 20 males and 20 females, aged 20–75 years (mean 56.4 ± 14.2 years). The etiologies comprised 24 cases of malignant melanoma, 3 cases of melanocytic nevus, and 13 cases of refractory wounds (including chronic ulcers and sinuses). The defect locations were: heel (21 cases), arch (4 cases), and forefoot (15 cases). The horn-shaped perforator pedicled flaps used ranged in size from a maximum of 13 cm × 7 cm to a minimum of 7 cm × 3 cm (mean flap size 9.8 × 5.2 cm). In two cases, minor distal wound dehiscence and necrosis were observed, which subsequently healed with full-thickness skin grafting and dressing changes, without affecting the ultimate clinical outcome. All other flaps achieved complete survival, with satisfactory functional and contour restoration.

The study received approval from the Clinical Research Ethics Committee of the First Affiliated Hospital of Anhui Medical University, and written informed consent was secured from all patients. It focused on adult patients aged 18 years and above, examining variables including age, gender, etiology, wound location, wound size, complications, and surgical outcomes (Table 1).

Continuous variables are presented as mean ± standard deviation (SD) or range. Categorical variables are presented as frequencies and percentages. Subgroup analyses were performed comparing outcomes between: (1) heel (*n* = 21) vs. forefoot (*n* = 15) vs. arch (*n* = 4) defects; (2) malignant melanoma (*n* = 24) vs. refractory wounds (*n* = 13) vs. benign nevi (*n* = 3). The association between defect size and complication rate was analyzed using an independent *t*-test. All statistical analyses were performed using SPSS version 26.0 (IBM Corp., Armonk, NY, USA), with statistical significance defined as *p* < 0.05.

### 2.2. Outcome Measures

Primary outcome: Flap survival, classified as complete survival, partial necrosis (requiring additional procedure), or total failure.

Secondary outcomes:

Functional outcomes assessed using the American Orthopaedic Foot and Ankle Society (AOFAS) Ankle–Hindfoot Scale at final follow-up (scores: 90–100 = excellent, 75–89 = good, 50–74 = fair, <50 = poor).

Sensory recovery evaluated using Semmes–Weinstein monofilaments (SWM) at the flap center, edge, and contralateral normal side. Protective sensation was defined as perception of 5.07/10 g filament or less. Two-point discrimination was measured using a standard Disk-Criminator.

Aesthetic outcomes rated by two independent plastic surgeons (blinded to the study) using a 5-point Likert scale (1 = poor, 2 = fair, 3 = good, 4 = very good, 5 = excellent) for color match, contour, and scar appearance.

Time to full weight-bearing, defined as the postoperative week when the patient could ambulate without assistive devices without wound breakdown.

Oncologic outcomes for melanoma patients: Breslow thickness, ulceration, pathological stage (AJCC 8th edition), margin status, local recurrence, and distant metastasis.

### 2.3. Surgical Technique

#### 2.3.1. Flap Design

Preoperatively, a Doppler flowmeter was employed to identify and mark at least 2–3 prominent superficial pulsation points within the perforator-rich zones around the plantar defect. Taking the defect site as the leading edge (with a width exceeding the defect diameter), a horn-shaped perforator-based flap was designed. Preoperative planning checklist: Doppler mapping: Identify and mark ≥ 2 perforators within 2 cm of defect edge. Defect measurement: Record maximum diameter (cm). Flap width at leading edge: Exceed defect diameter by 0.5–1 cm. Flap length: 3–4× defect length. Flap orientation: Tail directed toward medial non-weight-bearing arch. Perforator inclusion: Ensure at least 2 perforators lie within the designed flap territory. For heel defects: Tail extends posteromedially. For forefoot defects: Tail extends posteromedially along the arch. For arch defects: Bilateral propellers considered if defect > 4 cm

#### 2.3.2. Flap Elevation

**Figure 1:**
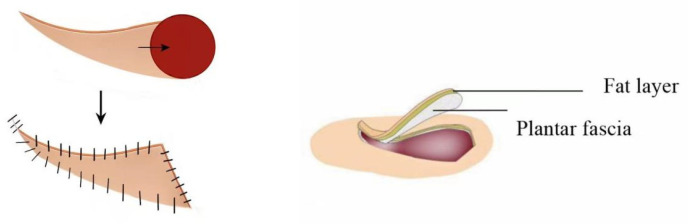
“Angular” design and flap harvesting plane.

The lesion was excised with a margin of 0.5–1 cm from the wound edge (extended to 1.5–2 cm for malignant tumors), reaching the superficial layer of the calcaneal periosteum. The flap was incised along the marked lines and its edges were raised. Sharp dissection was carried out down to the subcutaneous fascial layer. The plantar aponeurosis could be detached from its attachments at the calcaneus and metatarsals and elevated along with the flap. To optimize the rotation–advancement distance of the propeller flap while safeguarding the major perforators, the deep source vessels supplying the flap (providing primary and secondary blood supply) could be managed. For instance, when repairing a heel pad defect, the perforating branch of the lateral plantar artery (a secondary source) could be ligated and divided (done in three cases in this series, including one case of accidental intraoperative injury and ligation), with the medial plantar artery preserved as the primary pedicle. At the tail end of the horn-shaped flap, the subcutaneous branches of the medial plantar vessels emerging superficial to the abductor hallucis muscle, as well as the small perforators extending to the medial arch and dorsum, were all divided. This ensured the subcutaneous tissue pedicle was less than one-third of the flap length, facilitating rotation–advancement towards the heel pad or forefoot (Figure 1). Clarification of flap biomechanics: In this series, the flap rotation angle ranged from 45° to 90° (mean 67°), which is less than the 90–180° rotation typically required for a “propeller flap” as defined by the original Pirotta classification. Therefore, our flap functions predominantly as an advancement–rotation flap rather than a true propeller flap. A single dominant perforator was not routinely skeletonized; instead, 2–3 perforators were preserved within a broad subcutaneous pedicle to maximize perfusion reliability.

#### 2.3.3. Standardized Surgical Algorithm

① Perforator selection criteria: Primary perforator: Largest caliber (>0.5 mm on Doppler), located within 2 cm of defect edge, with audible signal. Secondary perforators: Any additional signals within the designed flap territory. At least 2 perforators must be included; if only 1 is identified, consider alternative flap design.

② Management of perforators: Preserve all identified perforators within the flap pedicle. Ligation of the lateral plantar perforating branch (secondary source) is permissible in selected cases (performed in 3/40 cases, 7.5%) without flap compromise. Do not ligate the medial plantar artery or its main perforators.

③ Maximum safe rotation angle: Based on our clinical experience, the safe rotation range is 45–90°. Rotations > 90° significantly increase risk of pedicle torsion and venous congestion; for such cases, consider bilateral flap design or alternative reconstruction.

④ Intraoperative tension assessment: After flap inset, assess capillary refill (<3 s) and skin blanching. If refill > 3 s or persistent blanching, consider: (1) further deep fascia release, (2) triangular flap interdigitation (Option 1 below), or (3) conversion to bilateral flap design.

#### 2.3.4. Wound Reconstruction

After full flap elevation, the flap was rotated and advanced into the defect. The wide leading edge of the flap, with its two prominent cup-shaped triangular extensions, could be handled flexibly in cases of significant tension or limited advancement distance:

① Under high tension, the triangular flaps could be interdigitated with the wound edges in a “Z”-plasty fashion. This reduced tension and minimized scar contracture compared to linear closure.

② When advancement was limited, the triangular flaps could be sutured together in a “kiss” fashion (direct apposition), effectively lengthening the flap.

**Figure 2:**
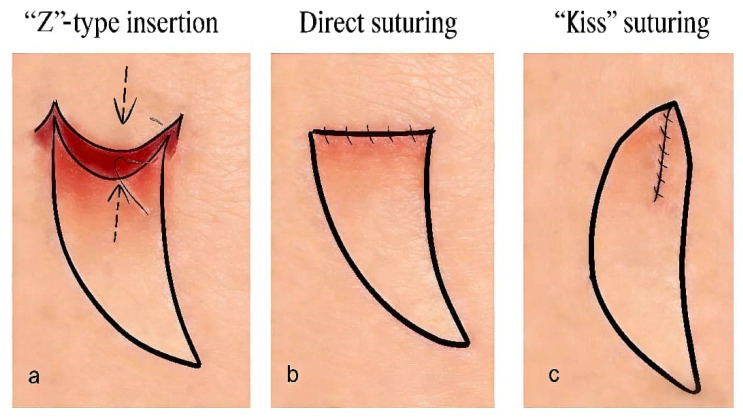
Flexible application of the triangular skin flap at the leading edge. (**a**) ”Z”-type insertion. (**b**) Direct suturing. (**c**) “Kiss” suturing.

③ Under minimal tension, the edges of the triangular flaps could be trimmed and the wound closed primarily with direct apposition (Figure 2).

During suturing, care was taken to avoid deep stitches that might inadvertently ligate the underlying perforators and compromise flap perfusion. The resulting donor-site defect was closed primarily using layered sutures.

#### 2.3.5. Results

① Flap Survival and Complications.

In this series, 38 of the 40 flaps survived completely (95%). Minor distal wound dehiscence and necrosis occurred in two cases, but these issues resolved successfully following full-thickness skin grafting and routine dressing changes, without negatively affecting the final functional and aesthetic results. All patients experienced satisfactory restoration of both plantar contour and function (Table 2).

② Statistical Analysis Results.

The mean patient age was 56.4 ± 14.2 years (range 20–75). Mean defect size was 3.4 × 2.7 cm (range 2 × 1.5 cm to 5 × 5 cm). Mean flap size was 9.8 × 5.2 cm (range 7 × 3 cm to 13 × 7 cm). The overall complete flap survival rate was 95.0% (38/40). The two cases with partial distal necrosis (5.0%) had defect sizes of 4 × 3 cm and 3 × 2 cm, respectively; no statistically significant association was found between defect size and complication occurrence (*p* = 0.67). Subgroup analysis revealed no significant difference in flap survival between heel (95.2%, 20/21) and forefoot (93.3%, 14/15) defects (*p* = 0.82). Malignant melanoma cases (*n* = 24) had a 95.8% complete survival rate, compared to 92.3% (12/13) for refractory wounds (*p* = 0.65). These comparisons are limited by the small number of complications.

③ Functional and Sensory Outcomes.

At a mean follow-up of 14.2 ± 6.8 months (range 3–24 months), the mean AOFAS score was 91.3 ± 5.6 (range 78–100), with 32 patients (80%) achieving excellent scores and 8 patients (20%) good scores. No patient had fair or poor functional outcomes.

Sensory testing with Semmes–Weinstein monofilaments was performed in 31 patients (77.5%) at final follow-up. Protective sensation (≤5.07/10 g) was present in 27 patients (87.1%). Mean two-point discrimination was 12.3 ± 3.1 mm (range 8–18 mm) at the flap center, compared to 9.8 ± 2.4 mm at the contralateral normal heel (*p* = 0.003).

Aesthetic scores from independent reviewers: Mean overall score 4.2 ± 0.6 (range 3–5), with color match 4.3 ± 0.7, contour 4.1 ± 0.8, and scar appearance 4.0 ± 0.7.

The mean time to full weight-bearing was 6.4 ± 1.8 weeks (range 4–12 weeks). No patient experienced ulcer recurrence or wound breakdown after achieving full weight-bearing during the follow-up period.

④ Oncological Outcomes (Malignant Melanoma, *n* = 24) (Table 3).

For benign nevi (*n* = 3) and refractory wounds (*n* = 13), no oncologic surveillance was required. No malignant transformation was observed in the chronic wound cohort.

⑤ Postoperative care.

Postoperative care consists of close monitoring of flap perfusion, prevention of pedicle compression, and early recognition of venous congestion or arterial insufficiency. Key measures include limb elevation, avoidance of external pressure, adequate hydration and nutrition, and prophylactic antibiotics when indicated. Patients should keep the surgical site clean and dry, avoid movements or dressings that might kink the pedicle, and promptly report signs of infection such as increased pain, erythema, or discharge. Gradual supervised mobilization, smoking cessation, regular wound checks, and follow-up visits at 1, 2, and 4 weeks post-discharge are recommended to assess flap healing and donor site recovery.

## 3. Representative Cases

- Case 1

**Figure 3:**
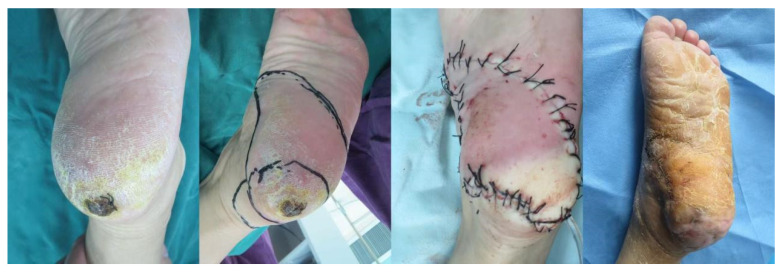
**Case 1.**

A 71-year-old female presented with a mass, roughly 3 cm × 2 cm in size, on her left heel. The planned excision margin was set to extend about 1 cm beyond the lesion. After using a Doppler flowmeter to pinpoint the surrounding arterial perforators, a propeller perforator flap with a length-to-width ratio of approximately 3.5:1 was designed (the flap area included two arterial perforators). Sutures were removed 3 weeks postoperatively, and the flap showed excellent survival. The pathological diagnosis confirmed malignant melanoma. At the 6-month follow-up, the patient had resumed a normal walking gait. The reconstructed area was well-healed and stable, with no signs of tumor recurrence (Figure 3).

- Case 2

**Figure 4:**
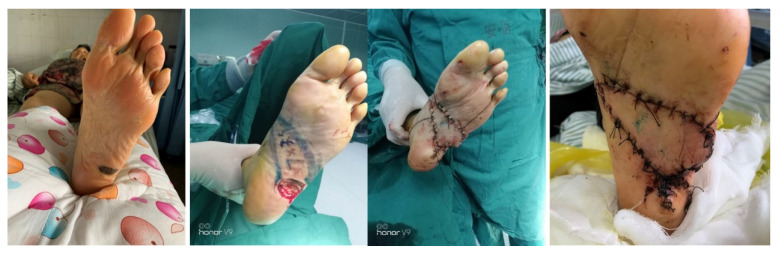
**Case 2.**

A 72-year-old female had a mass, roughly 3 cm × 2 cm, on the left plantar area. A Doppler flowmeter was used to identify the surrounding arterial perforators, based on which a pedicled propeller perforator flap with a length-to-width ratio of about 3.5:1 was designed (the flap area encompassed two arterial perforators). Intraoperative frozen section analysis after lesion excision revealed malignant melanoma with a base depth of [X] cm. The excision was then extended by an additional 1 cm. The flap was incised and elevated as planned. Sutures were taken out 3 weeks postoperatively, and the flap survival was outstanding. The final pathological diagnosis confirmed malignant melanoma. At the 6-month follow-up, the patient had resumed a normal walking gait. The reconstructed site was well-healed and stable, with no signs of tumor recurrence (Figure 4).

- Case 3

**Figure 5:**
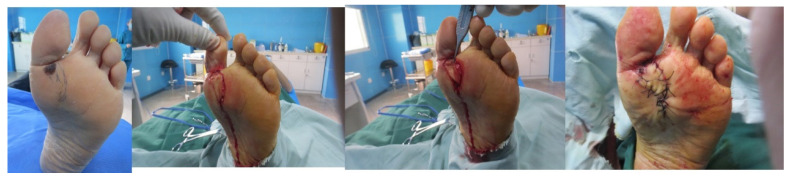
**Case 3.**

A female patient had a pigmented nevus, roughly 2 cm × 2 cm in size, on the anterior plantar area. After excision, the remaining wound was also 2 cm × 2 cm and could not be closed directly. A pedicled propeller perforator flap with a length-to-width ratio of 3:1 was then designed, incised and elevated accordingly. Sutures were removed 3 weeks postoperatively, and the flap showed excellent survival. The pathological diagnosis was junctional nevus. At the 6-month follow-up, the contour of the reconstructed area was normal, and it was well-healed and stable (Figure 5).

## 4. Discussion

The ideal reconstruction of the plantar region should restore its weight-bearing capacity, provide durable keratinized skin, and recreate the normal anatomical contour of the plantar surface [7]. Various options exist for plantar reconstruction, such as secondary wound healing, skin grafts, or free flaps [8]. Partial amputation in young patients should be avoided whenever possible [9]. The “propeller flap” was first introduced and applied by Donnell in 1992 for facial defect reconstruction. Its name derives from its resemblance to a rhinoceros horn and its rotational advancement mechanism [10]. In 2003, Georgeu et al. successfully used this technique to reconstruct skin defects after malignant tumor resection on the lower leg, reporting 24 cases [11], though they did not specify the pedicle’s blood supply. The clinical application of perforator flaps was first reported by Stepanov in 1980 [12]. In 1988, Kroll further defined the “perforator-based flap” as a flap relying solely on a perforating vessel, with only skin and subcutaneous tissue retained [13]. This highlights it as an axial pattern flap centered around a perforator vessel. By integrating the “propeller” design with the perforator-based flap, we proposed the perforator-based propeller flap, which includes a perforator pedicle and part of the fascia. We applied this flap to reconstruct small-to-medium-sized plantar defects, offering a new approach for plantar wound repair. Traditional distant axial flaps risk pedicle compression during transfer, potentially compromising flap perfusion [14]. Our flap, relying on local perforator vessels for blood supply, has a pedicle in the middle third of the flap, providing a “vertical blood supply” (Figure 4) for more reliable perfusion. Since it carefully preserves perforators within the flap, its blood supply is superior to that of local random-pattern flaps. Compared to traditional axial flaps, its harvest does not sacrifice major named arteries or veins, thereby reducing the risk of postoperative venous congestion [15].

The anatomical basis of the medial plantar vascular system has been studied [16]. The posterior tibial artery arises from the distal intermuscular septum between the abductor hallucis and flexor digitorum brevis muscles. Below the abductor hallucis, it divides into a superficial and a deep branch at the talonavicular joint level, with a main trunk length ranging from 2.5 to 3.5 cm. Each of these branches, along with the medial branch, gives off perforators to the skin region, which are crucial for our anastomotic flap surgical strategy.

As the sole is a weight-bearing area with dense skin, the plantar propeller perforator flap, using native plantar skin, better matches the texture of the defect site compared to traditional medial plantar flaps [17]. Incorporating and advancing the plantar aponeurosis with the flap increases the thickness of the heel pad defect area, enhancing pressure tolerance and durability [18]. If accompanying nerves are preserved, sensory recovery is also promoted. Nourished by perforating vessels, the flap avoids injury to major arteries and benefits from multiple anastomoses, ensuring a robust blood supply. The propeller design allows for rotation–advancement, providing greater reach and movement freedom compared to the simple parallel advancement of a V-Y flap, increasing the potential repair area and reducing postoperative scar contracture. The donor site, shaped into a slender tail at the non-weight-bearing medial arch of the foot, is under minimal tension and can be closed primarily without skin grafting, avoiding morbidity at a new donor site [19].

During flap harvest, it is essential to preoperatively map the surrounding perforators using a Doppler flowmeter and mark them, ensuring the flap design includes 2–3 larger perforators. The leading edge of the flap should be wider than the defect diameter, with a length at least three times that of the defect. The ratio of pedicle length to total flap length should be approximately 1:3 to 1:4. Dissection should be close to the deep fascia, preserving the plantar aponeurosis to protect the perforators or the subcutaneous pedicle within the flap and enhance flap thickness and durability. If excessive tension occurs during rotation–advancement, incise the deep fascia on both the convex and concave sides of the flap, preserving only the neurovascular perforators. Given the dense, thick, and poorly elastic nature of plantar skin, the unilateral perforator-based propeller flap is suitable for small-to-medium-sized plantar defects. For defects larger than 4 cm in diameter, it is advisable to flexibly use the combined advancement of adjacent propellers or local flaps to expand the clinical indications of this technique. Moreover, the sample size for plantar reconstruction in this study is relatively small; future research should include a larger sample size with regular follow-up [20].

To contextualize our results, we compared our technique with established alternatives (Table 4).

Beyond the reconstructive technique itself, this study points toward a future where image-recognizing artificial intelligence could standardize and objectify postoperative flap monitoring, as recent studies have demonstrated strong performance of AI systems in skin image interpretation [21]. Successful outcomes with the horn-shaped perforator flap depend heavily on careful visual assessment—from preoperative lesion evaluation to postoperative monitoring for flap viability, necrosis, dehiscence, and recurrence. Image-recognizing AI could help standardize documentation, quantify healing trajectories, and enable earlier recognition of subtle complications or recurrence signals that might otherwise rely on subjective interpretation. AI would not replace surgical judgment but could augment this reconstructive approach by making surveillance more objective, reproducible, and scalable. Future prospective studies should consider incorporating standardized digital photographic protocols with AI-based wound assessment, automated perfusion measurements, and machine learning algorithms to predict flap complications.

## 5. Conclusions

In conclusion, this study confirms that the horn-shaped perforator flap is a reliable and effective surgical option for repairing small-to-medium-sized defects (2–6 cm diameter) on the sole. With a 95% complete flap survival rate, excellent functional recovery (mean AOFAS 91.3), protective sensation in 87% of patients, and primary donor-site closure without skin grafting, this technique offers significant advantages in both functional and aesthetic restoration of soft tissue defects in weight-bearing areas of the foot. The technique is reproducible when the standardized algorithm is followed (Doppler mapping of ≥2 perforators, flap length 3–4× defect length, preservation of multiple perforators, rotation 45–90°, and flexible triangular leading-edge closure). However, prospective comparative studies with standardized plantar-specific outcome measures (including long-term durability > 2 years, validated functional scores, and objective sensory testing) are needed to define the optimal indications and to compare this technique with medial plantar flaps, free flaps, and dermal substitutes. In appropriately selected cases—small-to-medium plantar defects with intact adjacent perforators—the horn-shaped perforator flap is worthy of broader application.

## Figures and Tables

**Table 1:** Patients’ conditions.

No.	Age/Sex	Cause	Defect Site	Size of the Defect	Complications	Reconstruction Outcome
1	54/F	Malignant melanoma	heel	5 × 4	No	Excellent
2	36/M	Chronic ulcer	heel	4 × 3	No	Excellent
3	38/M	Chronic ulcer	heel	3 × 3	No	Excellent
4	42/F	Malignant melanoma	heel	5 × 4	No	Good
5	55/M	Malignant melanoma	heel	4 × 3	No	Excellent
6	57/M	Chronic sinus	front planta	3 × 2	No	Excellent
7	61/M	Malignant melanoma	front planta	5 × 3	No	Excellent
8	38/M	Chronic ulcer	arch of foot	4 × 3	No	Excellent
9	68/F	Chronic ulcer	front planta	5 × 2	No	Excellent
10	62/M	Malignant melanoma	heel	4 × 3	No	Excellent
11	72/F	Malignant melanoma	front planta	4.5 × 2	No	Excellent
12	54/M	Malignant melanoma	heel	3 × 2	No	Distal end necrosis
13	44/F	Malignant melanoma	front planta	2 × 1.5	No	Excellent
14	54/F	Pigmented nevus	heel	2 × 2	No	Excellent
15	67/F	Malignant melanoma	heel	3 × 2	No	Excellent
16	72/M	Pigmented nevus	front planta	3 × 3	No	Excellent
17	21/M	Chronic ulcer	front planta	2 × 1.5	No	Excellent
18	23/F	Pigmented nevus	heel	3.5 × 2	No	Excellent
19	65/M	Malignant melanoma	front planta	2 × 1.5	No	Excellent
20	42/M	Chronic ulcer	heel	3.5 × 2.5	No	Excellent
21	70/F	Malignant melanoma	heel	3 × 2.5	No	Excellent
22	49/F	Malignant melanoma	front planta	3 × 2	No	Excellent
23	75/F	Chronic ulcer	heel	2 × 2	No	Excellent
24	62/M	Malignant melanoma	heel	4 × 4	No	Excellent
25	71/M	Malignant melanoma	front planta	5 × 4	No	Excellent
26	72/M	Malignant melanoma	heel	3 × 3	No	Good
27	56/F	Malignant melanoma	arch of foot	3 × 3	No	Excellent
28	54/F	Chronic ulcer	front planta	4 × 3	No	Distal end necrosis
29	51/F	Malignant melanoma	heel	3.5 × 2	No	Excellent
30	73/F	Malignant melanoma	front planta	3 × 2	No	Excellent
31	70/M	Malignant melanoma	front planta	2.5 × 2	No	Excellent
32	66/F	Chronic ulcer	heel	3 × 2	No	Excellent
33	60/M	Malignant melanoma	front planta	4 × 4	No	Excellent
34	71/M	Chronic ulcer	heel	3 × 3	No	Excellent
35	71/F	Malignant melanoma	heel	3 × 2	No	Excellent
36	62/F	Malignant melanoma	heel	5 × 5	No	Excellent
37	59/M	Chronic ulcer	arch of foot	4 × 4	No	Excellent
38	62/F	Malignant melanoma	heel	4 × 4	No	Excellent
39	67/F	Malignant melanoma	arch of foot	2.5 × 1.5	No	Excellent
40	70/M	Chronic ulcer	front planta	2 × 2	No	Excellent

**Table 2:** Flap survival and clinical outcomes.

Parameter	Result
Total Cases	40
Complete Flap Survival	38 (95%)
Partial Complication	2 (5%)
Complication Description	Minor distal wound dehiscence and necrosis
Management	Full-thickness skin grafting and dressings
Final Outcome	Healed without compromising function/contour
Functional & Aesthetic Outcome	Satisfactory restoration in all cases

**Table 3:** Oncological outcomes.

Parameter	Value
Mean Breslow thickness (mm)	2.4 ± 1.2 (range 0.8–5.5)
Ulceration present	9 (37.5%)
Pathological stage (AJCC 8th)	
—Stage I	11 (45.8%)
—Stage II	8 (33.3%)
—Stage III	5 (20.8%)
Sentinel lymph node biopsy performed	18 (75.0%)
—Positive nodes	4 (16.7%)
Surgical margins	
—Negative (R0)	24 (100%)
—Margin distance (mm)	12.5 ± 3.8
Local recurrence during follow-up	0 (0%)
Distant metastasis	1 (4.2%)—lung at 18 months
Mean oncologic follow-up (months)	16.3 ± 5.7 (range 6–24)

**Table 4:** Comparison with alternative reconstructive options.

Technique	Advantages	Disadvantages	Best Indication
Horn-shaped perforator flap (our technique)	Glabrous skin match, preserves major vessels, primary donor closure, combined rotation–advancement	Limited to defects < 6 cm, requires intact adjacent perforators, learning curve	Small-to-medium plantar defects (2–6 cm)
Medial plantar (instep) flap	Excellent glabrous skin match, reliable anatomy	Sacrifices medial plantar artery, shorter reach (5–6 cm), donor graft often needed	Moderate defects with named vessel sacrifice acceptable
Free flaps (ALT, radial forearm)	Large volume, long reach, versatile	Microsurgical expertise, longer OR time (4–6 h), 5–10% failure rate, texture mismatch	Large defects > 6 cm, complex 3D defects
Skin grafting	Simple, quick	Poor durability under load, ulceration risk, contour depression, no sensation	Non-weight-bearing areas, temporary coverage
Dermal substitutes	Useful for large wounds, reduces donor morbidity	Two-stage procedure, costly, regenerated skin lacks adnexa	Bridge to grafting, partial-thickness defects

## Data Availability

The datasets used and/or analyzed during the current study are available from the corresponding author on reasonable request.
